# Supplementary material for: Cloud BioLinux: pre-configured and on-demand bioinformatics computing for the genomics community
Source: BMC Bioinformatics. 2012 Mar 19;13:42. doi: 10.1186/1471-2105-13-42 (PMC3372431; doi:10.1186/1471-2105-13-42)
Supplement: Additional file 1 — Supplementary 1 Cloud BioLinux software documentation in the form of a mini, self-contained website. Users need to download and uncompress the .zip file, and open through a web browser the "index.html" file available on the main directory. (ZIP 1823 kb). [file 1471-2105-13-42-S1.ZIP › Cloud-BioLinux-Package-Documentation/docs/biojava.html]

Bio-Linux Software Documentation Pages

Back to search form

## biojava

|  |  |
| --- | --- |
| Name | biojava |
| Description | BioJava is an open-source project dedicated to providing a Java framework for processing biological data. It include objects for manipulating sequences, file parsers, DAS client and server support, access to BioSQL and Ensembl databases, and powerful analysis and statistical routines including a dynamic programming toolkit. |
| Homepage | http://biojava.org/wiki/Main\_Page |
| Remote Documentation |  |
